# Acknowledging uncertainty about maternal mortality estimates

**DOI:** 10.2471/BLT.15.155036

**Published:** 2015-11-25

**Authors:** Rob E Dorrington, Debbie Bradshaw

**Affiliations:** a Centre for Actuarial Science (CARe), University of Cape Town, Private Bag, Rondebosch, Cape Town, 7700, South Africa.; b Burden of Disease Research Unit, South African Medical Research Council, Tygerberg, South Africa.

Differences between estimates of maternal mortality from different research groups have been much debated.^1^^–^^3^ The data that are included or omitted and the choice of models have been discussed, but the uncertainty of the estimates has not received much attention. Attempts to estimate the maternal mortality ratio (MMR) for South Africa published in the past five years illustrate the large variability of estimates and the tendency to underestimate uncertainty, particularly when MMR is low.

Fig. 1 shows the three most recent estimates of MMR published by the Institute for Health Metrics and Evaluation (IHME)^4^^–^^6^ and the two most recent estimates published by the World Health Organization (WHO).^7^^,^^8^ In addition, two sets of estimates derived from local data are shown: (i) the confidential enquiry based on maternal deaths recorded in health facilities and births at those facilities;^9^ and (ii) the indicator used by the Health Data Advisory and Coordination Committee (HDACC) of the Department of Health.^10^ The latter is based on registered maternal deaths from vital registration, corrected for general incompleteness of reporting of death plus 50% to allow for maternal deaths incorrectly attributed to other causes.^11^ Neither of the estimates based on local data purports to produce an accurate estimate of maternal mortality, but both attempt to track changes over time on a reasonably consistent basis.

**Fig. 1:**
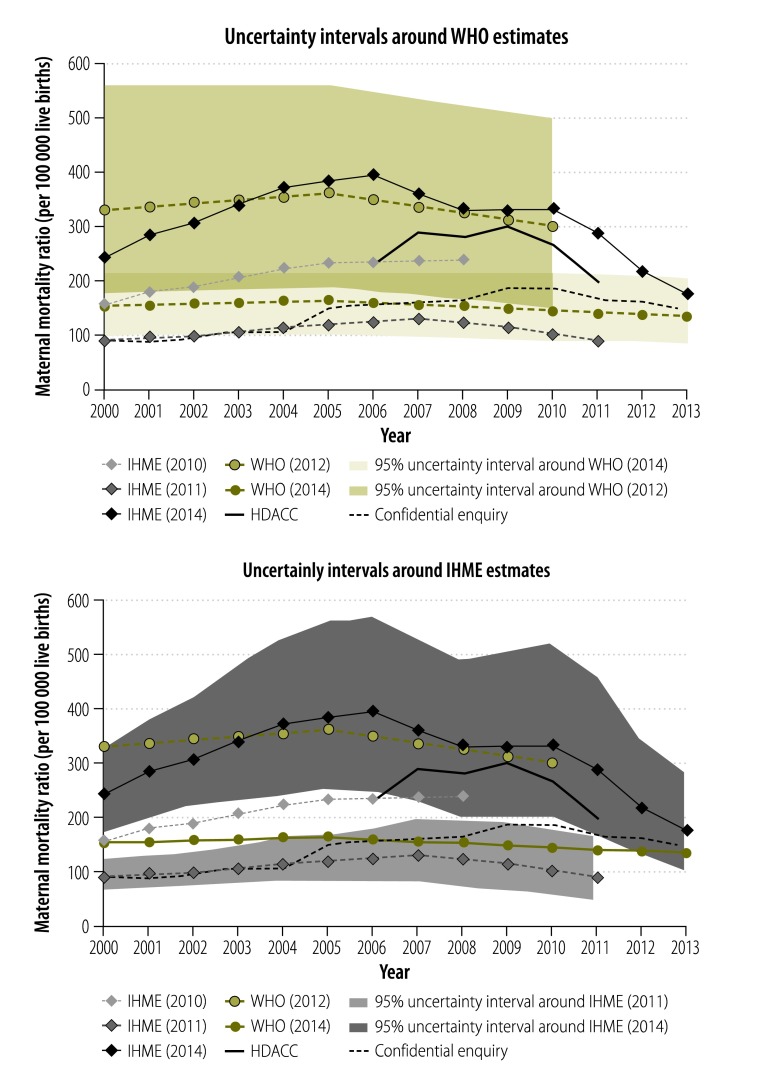
Estimates of the maternal mortality ratio for South Africa from several data sources, 2000 to 2013

HDACC: Health Data Advisory and Coordination Committee of the Department of Health; IHME: Institute for Health Metrics and Evaluation; WHO: World Health Organization.

Fig. 1 includes the 95% uncertainty intervals about the WHO estimates and Fig. 2 the 95% uncertainty intervals about the IHME estimates. These intervals supposedly indicate the uncertainty about the estimates. The most recent estimates from IHME and WHO lie outside the respective 95% uncertainty intervals about their previous estimates. In the case of IHME, even though the most recent estimates acknowledge a much greater degree of uncertainty, the uncertainty intervals don’t overlap those about the immediately preceding estimates. The most striking feature of these estimates is the wide range spanned by the WHO and IHME estimates. The second feature is the difference between the most recent and second most recent published estimates. The third is that where the IHME estimates now lie above the other estimates, the WHO estimates lie below the others, in contrast to their previous estimates.

Unless a country has a nationally representative system to track maternal deaths, there is likely to be a great deal of uncertainty about maternal mortality. Currently this uncertainty is not being fully acknowledged by those attempting to estimate MMR indirectly, particularly as part of efforts to track maternal mortality globally. This, together with the fact that estimates of MMR can change significantly from one set of estimates to the next, make them useless for informing countries about what is or is not working to combat maternal mortality.

Countries should monitor the effectiveness of policy and interventions using an index for tracking changes over time, such as the institutional MMR, even if this does not necessarily produce an accurate estimate of the absolute level of the maternal mortality in the country. The global initiatives that produce country-specific estimates should allow for greater model uncertainty and balance in-depth evaluation of country-specific data against finding one model that fits all.

Given that the target 3.1 of the Sustainable Development Goals is to reduce the global maternal mortality ratio to less than 70 per 100 000 live births by 2030,^12^ maternal mortality will be a key indicator to measure target progress. Therefore, investment is needed in civil registration and vital statistics for improved measurement of maternal mortality by low- and middle-income countries.^13^

## References

[1] AbouZahr C. New estimates of maternal mortality and how to interpret them: choice or confusion? Reprod Health Matters. 2011 5;19(37):117–28. 10.1016/S0968-8080(11)37550-7 21555092

[2] Horton R. Maternal mortality: surprise, hope, and urgent action. Lancet. 2010 5 8;375(9726):1581–2. 10.1016/S0140-6736(10)60547-8 20382418

[3] Kassebaum NJ, Lopez AD, Murray CJL, Lozano R. A comparison of maternal mortality estimates from GBD 2013 and WHO. Lancet. 2014 12 20;384(9961):2209–10. 10.1016/S0140-6736(14)62421-1 25625393

[4] Kassebaum NJ, Bertozzi-Villa A, Coggeshall MS, Shackelford KA, Steiner C, Heuton KR, et al. Global, regional, and national levels and causes of maternal mortality during 1990–2013: a systematic analysis for the Global Burden of Disease Study 2013. Lancet. 2014 9 13;384(9947):980–1004. 10.1016/S0140-6736(14)60696-6 24797575 PMC4255481

[5] Lozano R, Wang H, Foreman KJ, Rajaratnam JK, Naghavi M, Marcus JR, et al. Progress towards Millennium Development Goals 4 and 5 on maternal and child mortality: an updated systematic analysis. Lancet. 2011 9 24;378(9797):1139–65. 10.1016/S0140-6736(11)61337-8 21937100

[6] Hogan MC, Foreman KJ, Naghavi M, Ahn SY, Wang M, Makela SM, et al. Maternal mortality for 181 countries, 1980–2008: a systematic analysis of progress towards Millennium Development Goal 5. Lancet. 2010 5 8;375(9726):1609–23. 10.1016/S0140-6736(10)60518-1 20382417

[7] Trends in Maternal Mortality. 1990 to 2010: WHO, UNICEF, UNFPA and the World Bank Estimates. Geneva: World Health Organization; 2012. Available from: http://www.who.int/reproductivehealth/publications/monitoring/9789241503631/en/ [cited 2015 Nov 17].

[8] Trends in Maternal Mortality: 1990 to 2013. WHO, UNICEF, UNFPA, The World Bank and the United Nations Population Division. Geneva: World Health Organization; 2014. Available from: http://www.who.int/reproductivehealth/publications/monitoring/maternal-mortality-2013/en/ [cited 2014 Jun 7].

[9] Pattinson R, Fawcus S, Moodley J. Tenth interim report on the confidential enquiries into maternal deaths in South Africa 2011-12. Pretoria: National Committee for Confidential Enquiries into Maternal Deaths, Department of Health; 2013.

[10] Dorrington RE, Bradshaw D, Laubscher R, Nannan N. Rapid mortality surveillance report 2013. Cape Town: South African Medical Research Council; 2014. Available from: http://www.mrc.ac.za/bod/RapidMortalitySurveillanceReport2013.pdf [cited 2015 Jan 12].

[11] Trends in Maternal Mortality: 1990 to 2008: Estimates Developed by WHO, UNICEF, UNFPA, and the World Bank. Geneva: World Health Organization; 2010. Available from: http://whqlibdoc.who.int/publications/2010/9789241500265_eng.pdf [cited 2012 Mar 31].

[12] Sustainable Development Goals. New York: United Nations; 2015. Available from: https://sustainabledevelopment.un.org/topics [cited 2015 Nov 13].

[13] AbouZahr C, de Savigny D, Mikkelsen L, Setel PW, Lozano R, Lopez AD. Towards universal civil registration and vital statistics systems: the time is now. Lancet. 2015 10 3;386(10001):1407–18. 10.1016/S0140-6736(15)60170-2 25971217

